# A qualitative exploration of escalation of care in the acute ward setting

**DOI:** 10.1111/nicc.12479

**Published:** 2019-12-12

**Authors:** Jody Ede, Emma Jeffs, Sarah Vollam, Peter Watkinson

**Affiliations:** ^1^ Nuffield Department of Clinical Neurosciences University of Oxford Oxford England; ^2^ Emergency Department Royal Children's Hospital Melbourne Victoria Australia

**Keywords:** acute ward, deterioration, escalation of care, observations, qualitative

## Abstract

**Background:**

“Failure to Rescue” includes failing to prevent avoidable patient deterioration and death. Despite its use, delays in care escalation still affect patient outcomes.

**Aims and Objective:**

The aim of this qualitative service evaluation was to map the barriers and facilitators to the escalation of care in the acute ward setting and identify those that are modifiable.

**Design:**

A total of 55 hours of qualitative observations were completed to capture care escalation events. These were conducted at two hospital sites in one National Health Service trust.

**Methods:**

Observations were iterative, with research team meetings being used to discuss the data and future methods. Field notes were analysed thematically by two researchers, extracting data on barriers and facilitators to escalation of care.

**Results:**

Clinical nursing staff challenged the sensitivity and specificity of Early Warning Scores, describing tool failings in certain clinical scenarios. Staff did not escalate based on the alerting Early Warning Scores alone but used other clinical factors, such as bleeding, which are not necessarily captured in the scoring systems. Staff frequently did not re‐escalate low‐level scores. Patient and non‐patient factors identified as posing barriers to escalation were complex care needs, patient outlier status, and involvement of multiple care teams. Factors negatively affecting the chain of communication during escalation were team tension, staffing levels, and inadequate handover.

**Conclusion:**

This service evaluation identified barriers and facilitators to the escalation of care in the acute ward setting. Unlike other studies, we found that re‐escalation or tracking of deterioration was problematic. Patients identified as being at a higher risk of escalation failure included complex patients, outliers, and patients with multiple care teams.

**Relevance to clinical practice:**

This service evaluation demonstrates continuing health care communication barriers. Patient groups (complex patients and outliers) risk process failures during escalation. This can be applied in clinical practice by staff anticipating problems in these patients, documenting clear escalation pathways.

## 1 INTRODUCTION

Patients who die as a result of reversible complications are classified as a Failure to Rescue (FTR).^1^, ^2^, ^3^ Patients often have predictable abnormal vital signs hours before deterioration,^4^, ^5^ and an ability to quickly recognize and escalate deterioration affects FTR rates.^6^ Sentinel events are estimated to be 30% in low‐volume hospitals,^2^ and of harm incidents reported to the National Patient Safety Agency, 32% had issues of diagnostic errors and poor recognition of deterioration.^7^ FTR events result from problematic escalation of care,^8^ and understanding the facilitators and barriers to this are integral to designing safer health care systems.

**WHAT IS KNOWN ABOUT THIS TOPIC?**

- Failure to Rescue (FTR) is a widespread problem within health care.
- Interventions exist to minimize FTR, such as Early Warning Scores (EWS).
- Human factors known to affect the escalation of care process are communication, clinical culture, and decision‐making.
- To design safer health care systems, research must focus on these human factors that affect detection of deterioration or the decision to escalate.

**WHAT THIS PAPER ADDS?**

- Re‐escalation of low‐level EWS triggers may be problematic.
- Factors that are outside EWS are important cues in the decision to escalate and are not directly measured by the tools.
- EWS scores are interpreted by clinical staff, possibly indicating that scoring systems do not entirely compliment the way staff currently escalate.
- Groups of patients are at a higher risk of complications during the escalation of care process, such as complex patients, outliers, and patients with multiple care teams.
- Communication failures attributed to inadequate handovers, team tension, and staffing levels were problematic during care escalation.

## 2 BACKGROUND

### 2.1 Early Warning Scores

Early Warning Scores (EWS) are an intervention to reduce FTR events.^9^ Staff input physiological values for heart rate, respiratory rate, peripheral oxygen levels, and blood pressure, which are scored based on level of derangement.^4^, ^10^ Aggregate scores direct staff to the next step in care escalation,^11^ such as increasing frequency of physiological monitoring or a senior review.^4^, ^12^, ^13^, ^14^

The National Health Service (NHS) has widespread adoption of EWS systems,^15^, ^16^ but there is little evidence of these supporting a reduction in patient mortality,^17^ predicting cardio‐pulmonary arrests,^18^ or being consistent in terms of sensitivity or specificity.^14^ Despite not showing a significant improvement in reducing mortality, EWS systems that are not followed are linked to adverse sentinel events.^19^ Variability in systems' sensitivity and specificity (false positives/false negatives) may impact its adoption and trust by clinical staff, resulting in delayed or inappropriate escalation of care.^20^

### 2.2 Escalation of care

Escalation of care is the recognition of deterioration and communication to a senior medical or nursing colleague, resulting in a deterioration management plan.^21^ Barriers and facilitators to escalation can be contextual and involve organizational factors such as communication, clinical culture, and decision‐making.^2^, ^10^ EWS is cited by clinical staff as a way to facilitate deterioration communication and package information,^12^, ^22^ but care delays because of poor communication remain significant.^4^, ^23^ Referral is high risk, with significant errors likely to occur^24^, ^25^ and failures being directly attributable to incomplete information.^4^ In recognition of this problem, tools have been developed to improve communication during referral, such as Situation, Background, Assessment, and Recommendation (SBAR).^26^

Socio‐cultural factors contributing to escalation delays have also been identified, such as tension within the team,^22^ fear of being seen as not coping,^19^ or negative emotions such as anxiety of escalating.^27^ Referrer seniority can affect perceived credibility of escalation, with some studies suggesting that senior staff are more likely to obtain help by verbalizing concerns effectively.^28^ A proportion of missed deteriorations with improper use of EWS has been attributed to direct care team cohesiveness.^21^, ^27^

In some failed escalation cases, decision‐making, professional judgement, or situational awareness may also play a role, where staff ineffectually predict severity of the patient's clinical trajectory and therefore do not escalate appropriately.^12^, ^29^ EWS systems are not efficient in isolation at detecting and escalating care, and other contextual factors must be explored before a reduction in mortality may be seen.^10^, ^13^

## 3 METHODS

The aim of this qualitative service evaluation was to map the barriers and facilitators to escalation of care in the acute ward setting and identify those that are modifiable. A qualitative methodology was deemed an appropriate approach to capture the nuanced and contextual factors influencing FTR. Qualitative observations can yield rich, thick descriptions not influenced by recall bias and allow observation of factors influencing FTR not necessarily apparent or likely to be explicitly defined by clinicians who are familiar with the workings of a ward.

### 3.1 Ward observations

Qualitative observations (55 hours) were conducted on 12 different medical and surgical hospital wards within a major teaching hospital trust. Observations focussed on capturing escalation of care events. These hospital ward areas were sampled to increase the likelihood of capturing escalation of care events given the acuity of patients and ensured that any escalation variation between clinical areas could be observed. This work was defined as a service evaluation because it was set in one trust with the goal of improving understanding locally about escalation of care and care of the acute ward patient.

The research staff conducting the observations commenced the fieldwork sessions by introducing themselves to the ward nursing co‐ordinator. Ward routine was documented, as well as events that were considered to have the potential to influence timely and appropriate escalation of deteriorating patients. Field notes were extensively annotated and reflected on. Semi‐structured ad‐hoc interviews were conducted during the shift observed. These brief opportunistic conversations with clinical staff were structured around an observation guide that helped target the qualitative observations and clarify what the researcher was observing. The observation guide was flexible but generally focussed on the patient the nurse was most worried about and their awareness of who on the ward had an elevated EWS. When a patient score was elevated, but the nurse allocated to him or her did not express clinical concern, this was also briefly explored through ad‐hoc semi‐structured interviews. These aimed to add detail to observed events, and answers were annotated into field notes. The ad‐hoc interview is a common technique in qualitative observational research and ethnographic research and can assist the researcher in triangulating the data and clarifying what they are observing.^30^ Observations were iterative, with the subject of interest (ward, patient, staff) changing reflexively depending on patient acuity. Field notes were written and dictated post‐observations episode. No patient details were collected, and all field notes were anonymized. Research team meetings discussed data and informed future methods of enquiry.

Data were transcribed and then analysed thematically by two researchers (E.J. and L.B.) using NVivo v7 software (2007, QSR International, United States). This was conducted iteratively and reflexively.^31^ Results were presented and discussed until consensus was reached. Using a grounded theory approach, the data were open coded. All text was coded under the dominant theme(s) present in a passage of text; axial coding then took place where relationships were determined between these themes, and then, selective coding was used to further explore themes of high relevance to the research question.^31^ The research team was asked by the research lead to be transparent about any possible bias during team meetings, and the researchers agreed that data saturation was reached when no new codes emerged during analysis.

#### 3.1.1 Analysis framework a priori

To understand the context of escalation of care, the research team decided that one of three key areas would have to be affected:

1. Timely clinical action
2. Recognition of patient deterioration
3. Appropriate treatment and management

The research team used this framework to search for instances in the clinical environment that could influence one of these three concepts.

#### 3.1.2 Reflexivity

The research team performing data collection and analysis were encouraged to discuss any biases or assumptions. The primary researcher is a Registered Nurse with acute care and research experience. The world view of the primary researcher was critical realism, which acknowledges that perception of reality can vary between individuals or groups.^32^ The research team had a mixture of clinical and non‐clinical backgrounds. They observed perceived barriers and facilitators to timely and appropriate recognition and escalation of clinical deterioration without interaction with individual patients. Education on the EWS was provided to non‐clinical research staff to enable them to understand the protocol for response to an elevated score.

#### 3.1.3 Rigour

A strategy to ensure rigour in qualitative research is systematic and self‐conscious research design, data collection, interpretation, and communication.^33^ Observations were conducted by a team of five researchers (E.J., B.L., D.V., J.D., C.H.), with varying clinical backgrounds (health care professionals, administrators, and a lay person). This promoted data credibility by ensuring that observations were undertaken by people with differing views and who may probe events differently, generating less‐biased field notes. One research team member was known to the ward areas and was encouraged to reflect on their assumptions in research team meetings; the other researchers were not known to clinical staff. To ensure dependability, observers had a data collection tool (observation guide) that explicitly detailed the area of interest. Confirmability was promoted with the use of extensively written field notes. Transferability was enhanced by observing areas of multiple specialities and using an multi‐disciplinary team for the qualitative observations.

### 3.2 Ethics

Considered a quality improvement initiative, this qualitative service evaluation was registered with the local research department and assigned Datix reference number: 3924. We have used the COREQ 32‐point checklist for reporting rigour. Verbal consent was obtained prior to observations, with participants having the option to decline and ask staff to move away from clinical situations. Posters and leaflets were made available to the ward staff. These contained an outline of the service evaluation aims and the methods for data collection, including the ward observations, and ad‐hoc interviews. There were also information sources for staff, such as the audit lead email and phone number to whom they could raise any concerns. If an undetected deterioration occurred, observers would have had a duty to report this deterioration to the correct clinical team, and practice would be escalated through clinical governance systems, but fortunately, this did not occur. No identifiable ward, staff, or patient details were recorded, and wards are referred to using an allocated research number or pseudonym.

## 4 RESULTS

A total of 55 hours of qualitative observations were conducted. From the observational data and ad‐hoc interviews, three main themes and six sub‐themes are presented. We present theme definitions in Table 1 and sub‐themes in Table 2.

**Table 1:** Definitions of observation themes and sub‐themes

Domain	Definition
Early Warning Scores (EWS)	The process for recording observations and escalating care
Patient and non‐patient factors affecting care decisions	Outside of early warning score, factors affecting patient care and the running of the ward
Chain of communication	Who is talking to whom and what are the barriers/facilitators to this happening

**Table 2:** Results of themes and sub‐themes observed relating to escalation of care

Domain	Themes
Early Warning Scores	Sensitivity and specificity
	Clinical response to Early Warning Scores
Patient and non‐patient factors affecting care decisions	Complex care (fluid management, comorbidities, and medical management)
	Outliers
	Involvement of multiple teams
Chain of communication	Tension, staffing, and inadequate handover

### 4.1 Early Warning Scores

The process of taking and recording observations was observed. Both nurses and health care assistants undertook observation measurements, with Health Care Assistants being more likely to undertake “bulk” observation sets. Registered Nurses tended to undertake observations on their own patients only rather than performing “bulk” rounds. Local practices using the EWS varied. For example, on one ward, a nurse was unaware about the high trigger score for her patient, and others justified inaction based on observations because “that is usual for the patient.” In contrast, on another ward, the co‐ordinator held a folder with all the patient EWS scores and documentation of what clinical action had been taken.

#### 4.1.1 Sensitivity and specificity of EWS

Nurses often referred to the non‐triggering patients who had other social/physiological/or psychological concerns, not captured by the tool. Many patient‐related factors determined patient acuity, indicating EWS lacked sensitivity. Nurses did not always feel concern about elevated EWS scores. Many observed situations demonstrated a lack of escalation protocol compliance. Triggering patients were not always re‐escalated if observations remained the “same level” of abnormal.

Ward 12: …discussed the [EWS]…from his experience…patients rarely trigger on the ward, but if they do he uses his clinical judgement as well as the [EWS] to assess the situation, he states he does not solely rely on the numbers generated and looks at the individual patient's history and current management.

Ward 4: …there are 2 oesophageal bleeds who are the most sick patients…not triggering because they are stable, but are the 2 patients with the highest potential for deterioration…

#### 4.1.2 Clinical response to EWS

For patients with high EWS scores, clinical interventions and nursing care was observed to match the acuity. Patients were specialled (provided with extra nursing resources), with staff being familiar with the patient's care plan.

Ward 1: Sickest patient on the ward is in bed 6, they're triggering a 14. The registrar is with him and also a senior nurse wearing a blue uniform. The senior nurse appears to be specialing the patient. The patient is attached to a DinaMap (vital signs monitoring machine)…

EWS were used as a factor in decision‐making, but the many other escalation decision‐makers were also considered of equal importance. The decision to attend to observations more or less frequently was not made with only the previously recorded EWS in mind and there were many instances of deviations from observation frequency recommendations.

Ward 12: I discussed the [EWS] with the male nurse… how he would manage the…patient. … from his experience so far patients rarely trigger on the ward, but if they do he uses his clinical judgement as well as the [EWS] to assess the situation, he states he does not solely rely on the numbers generated and looks at the individual patients history and current management.

Observations were sometimes reported in a tokenistic way to fulfil the obligations of the escalation pathway. When one of the staff nurses realized that we were interested in EWS, she tapped a registrar on the shoulder to “fill him in” on a few abnormal observations.

Ward 4: Low BP reported … no recommendations for treatment or reasons why. Indicates that clinical information sometimes handed over to “tick a box”…

### 4.2 Patient and non‐patient factors affecting care decisions

Nurses and medics cited patient and non‐patient factors that affected care decisions. Staff were concerned for patients who were not necessarily triggering but who had other clinical risk factors and also described contextual factors outside of the EWS system, which provided concern cues.

#### 4.2.1 Patient factors affecting care decisions

##### Fluid management, comorbidities, and medical management

Nurses recognized that care could improve in relation to fluid management, giving examples of poor recognition of dehydrated patients and inadequate record keeping. Clinical staff confusion was observed regarding medication prescribing, such as clarity of dose, resulting in a delay to medication administration.

WARD 7: …took over a patient who had only had 100mls of urine … in 11 hours, … patient was quite unwell …felt the thing that worked well…once she flagged up the problem…she had good communication between the team and the doctors acted on her concerns very quickly.

Ward 4: the student nurse hands over worrying obs to the registered nurses, low blood pressure, um. She then chases up the doctor for a plan…the patient's apparently for fluids. This has been written in the notes from the ward round…but hasn't been prescribed. The nurse has to go to a doctor from another team to prescribe the fluids because the team from the original ward round is unavailable.

Ward 10:… nurse is taking handover … they have a long conversation regarding a patient's insulin. … transferred a few days ago and there seems to have been a falling down in communication about what kind of insulin …, and what kind of regime they were on. It doesn't seem to have been picked up on transfer.

Patients with dementia and acute confusion required more intense staff observation. Similarly, patients with complex social or psychological histories required more nursing involvement and were believed by staff to have a greater potential to deteriorate than some patients triggering.

Ward 4: Discussion between medics and dietician about patient care. How to approach the “volatile and vulnerable patient”, who is “medically OK” but “not out of the woods”. Resource intensive patient despite not triggering – still has potential to be very sick.

#### 4.2.2 Non‐patient factors affecting care decisions

##### Outliers

Patients who were not on their “home ward” were observed to require excessive nursing input in liaising with their medical team. In one instance, the co‐ordinator on Ward 4 was observed to be calling three teams to obtain a medical team review for an outlier patient. Clinical staff expressed feelings that defining individual clinical responsibility for the patient (which person in which team) was challenging in these situations.

Ward 4: Patient identified by coordinator as having no medical review…calling 3 different medics to arrange a review of the patient…

Coordinator describes problem of outliers (patients who are not being looked after by the home team). Difficult to coordinate their care because no one wants to take responsibility for them.

##### Involvement of multiple teams

Complex patients with multiple sources of care and discharge planning required a significant amount of nursing and medical time, with staff being redirected in order to escalate a deteriorating patient's care. This was compounded by confusion about plans of care, miscommunication about discharge plans, and a lack of being able to contact teams using the methods described previously. In the medical notes review, this was also evident as teams were waiting for review from other teams in order to make decisions about care.

2300 Patient desaturated. Called Senior House Officer (SHO), told to call ICU, ICU told to call ENT, ENT didn't answer. ITU came at request of SHO. ENT consultant called by nurse, SHO told to review. ITU and ENT disagreeing about need for trachy”

### 4.3 Chain of communication

Interactions between clinical staff were frequently observed, and communication around patient care was considered by examining the flow of communication between different parties.

#### 4.3.1 Tension

Instances of disagreement and tension between staff were observed, potentially influencing the productivity of professional relationships.

Ward 4: Observed interaction between doctors and nursing staff – very tense. Doctor storms off. “I guess we'll agree to disagree.” …Would be difficult for someone not confident to escalate problems to someone who is very dismissive. When discussing patients, he is not giving eye contact and showing defensive postures.

#### 4.3.2 Staffing

Perceptions of inadequate staffing numbers, inadequate staff skill, and staff with limited scope of practice were identified very frequently during observations and interviews. Staff attributed this as a primary cause for delayed care, delayed discharges, and error. Staffing on some wards was also observed to be adequate, and staff commented how much easier their shifts were when this was the case.

Ward 7: if there was just one thing she would generally improve the ward it would be staffing. …last Saturday… she was the only permanent member of staff from that ward. There were 2 agency nurses and there was one member of staff who was familiar with the trust but wasn't familiar with the ward so she didn't know where anything was, she wasn't able to open doors cos she didn't know codes, she wasn't able to just do simple dressings without being told where everything was…

#### 4.3.3 Missed handover

There were instances where a nurse was off the ward attending to procedures/professional development, and they were not observed to handover their patients to anyone. Loss of information and disparate notes systems separated by profession about the patient's clinical conditions were also observed.

0930 The porters arrive to transfer that patient and the nurse leaves with them and the patient on a bed. She hands over to another nurse to say she is going to be off the ward but doesn't give any handover about the patients she's looking after.

## 5 DISCUSSION

This service evaluation mapped several themes influencing care escalation, including EWS, patient and non‐patient factors affecting patient escalation, and chain of communication. These themes have been divided into six sub‐themes, further expanding on the escalation of care process.

EWS sensitivity was challenged by staff who gave clinical examples. Despite being a protocol‐driven tool, staff exerted professional judgement while interpreting scores and actions, leading to inconsistency in escalation compliance^12^, ^34^ and the universal safety net of the tool being reduced.^12^ Variability in tool use presented as not complying with observation frequency (either increasing or decreasing from that of the protocol), not escalating to the correct person, and instances of escalation not occurring in any form. While there is a risk related to clinical interpretation of EWS scores, studies have conversely found that it is common for staff to detect deterioration using parameters that are outside of the EWS systems, sometimes before an appropriate alert has been generated.^35^ These parameters may have been identified in other studies to include “soft signals” of deterioration, such as changes to skin colour or breathing patterns.^22^ It could be argued that clinical judgement, relating to EWS interpretation, is a double‐edged sword, simultaneously reducing unnecessary escalations and hindering timely escalation. In this service evaluation, a number of qualitative observations captured correct responses to EWS, with patients allocated extra resources to match their acuity.

While the detection of patient deterioration is problematic in a number of escalation studies,^4^, ^36^ this service evaluation also found that the tracking of deterioration and re‐escalation can also be ineffective. There was evidence of staff not challenging the “status quo” of a patient's condition, possibly contributing to insidious events of deterioration. Influencing a culture of challenging assumptions and encouraging a level of meta‐cognition (facilitating clinician introspection on decisions and identifying assumptions) may be a feasible way to improve decision‐making in both nurses and clinicians during escalation. This method of awareness may feasibly be integrated into undergraduate or nurse training days.

Contextual factors affecting escalation outside of the EWS were consistent with the literature.^21^, ^22^, ^37^ Patient‐related factors were fluid balance needs and complex poly‐pharmacy,^10^, ^37^ and non‐patient‐related factors identified were outliers and patients who required multiple teams' inputs. Problems with fluid balance management that were identified in this service evaluation are consistent with other studies, including incomplete fluid balances.^10^ These fluid‐related problems have been suggested by studies to contribute to one‐third of surgical patient deaths.^38^ Poly‐pharmacy in complex patients is a risk factor for medication errors, including inappropriate doses, inappropriate drugs, side effects, drug interactions, and adverse drug events.^39^ Observed care delays in outliers and patients with multiple care teams were a result of confusion regarding plans, responsibilities, and delayed decisions. These factors may partially explain why there is evidence that outliers have higher readmission rates and length of stay.^40^, ^41^ There may be value in identifying high‐risk patients (complex patients, outliers, or patients with multiple teams) and mitigating this with a systematic approach to care, such as clear documentation or pre‐planned care escalation pathways.

Barriers to effective chain of communication, such as inadequate handover, variability in staffing levels, and tension between team members, were also observed.^42^, ^43^ Inadequate staffing has been linked to poorer patient outcomes in the literature.^44^ During the observations, staff described high workloads that resulted in reduced time spent with each patient, and there were concerns that junior staff were being expected to care for sick and deteriorating patients. Conversely, there were instances of adequate staffing, and staff commented how much easier their shifts were when this was the case. Communication failures impeded effective and efficient care,^45^ leading to delayed actions, tasks, and assumptions being made about the patient's conditions. Studies suggest that up to 30% of team communication is sub‐standard, resulting in failure,^46^ and is the root cause of 52% to 70% of adverse events.^47^

### 5.1 Limitations

The limitations of this service evaluation are that it was performed within the same trust and was aimed at understanding local practice. This makes results ungeneralizable to the wider NHS but can be used to prompt local service delivery improvements. Methodological rigour was a key focus during the design of the data collection strategies, but qualitative observations bring with them the possibility of researcher bias. Ethical considerations were carefully accounted for prior to data collection, such as the research team identifying an unwell patient (which had not already been detected or actioned), and this was mitigated by clear escalation strategies by the research team.

## 6 RELEVANCE TO CLINICAL PRACTICE

There remains variability in monitoring and escalation of deteriorating ward patients. This service evaluation has demonstrated that there continues to be significant communication barriers in health care and highlights groups of patients who are at greater risk of care process failures during escalation. This can directly be applied to clinical practice by encouraging staff to anticipate escalation in these types of patients before it is required and documenting clear escalation pathways. There needs to be a better understanding as to why variability to escalation occurs and how to bridge the gap between EWS and clinical judgement of patient deterioration risks demonstrated in this work.

## 7 CONCLUSION

This service evaluation has highlighted the complex and sometimes chaotic nature of patient escalation and the changing environment in which EWS operate. Despite protocols and EWS, clinical judgement continues to influence escalation of care. Although this may demonstrate appropriate prioritization in some cases, encouraging meta‐cognition methods may provide a feasible approach to minimizing the negative effects of this. These cues (challenging assumptions and encouraging meta‐cognition) could lead to a focus on local education or at a wider nurse training level. Unlike some escalation literature, this service evaluation found that re‐escalation of patients was more problematic than the initial detection of deterioration and that low‐level EWS were unlikely to be escalated.

## CONFLICT OF INTEREST

P.W. is the Chief Medical Officer for Sensyne Health. This company owns the license for System for Electronic Notification and Documentation (SEND) and has a research agreement with the University of Oxford.

## AUTHOR CONTRIBUTIONS

All authors had read and approved the final manuscript. P.W. gave clinical expertise to the project, and E.J. and S.V. were responsible for design of the project, data collection, and editing of the manuscript. J.E. was the primary author of the manuscript.

## Supporting information


**Appendix S1.** Supporting Information.
